# Effects of electroacupuncture for obesity: A protocol for systematic review and meta-analysis

**DOI:** 10.1097/MD.0000000000029018

**Published:** 2022-03-04

**Authors:** Junhyuk Kang, Koh-Woon Kim, Yeonho Seo, Mi-Yeon Song, Won-Seok Chung

**Affiliations:** a Department of Korean Medicine Rehabilitation, College of Korean Medicine, Kyung Hee University, Seoul, Republic of Korea; b Department of Korean Medicine Rehabilitation, Kyung Hee University Hospital at Gangdong, Seoul, Republic of Korea; c Department of Korean Medicine Rehabilitation, Kyung Hee University Medical Center, Seoul, Republic of Korea.

**Keywords:** electroacupuncture, meta-analysis, obesity, protocol, systematic review

## Abstract

**Background:**

Obesity—a public health problem that negatively affects the quality of life—is associated with various diseases, and its prevalence is on the rise. Although drugs and surgical interventions are used to treat obesity, they have adverse effects and limitations. Electroacupuncture is a widely used method for treating obesity in which electrical stimulation is transmitted to the body through acupuncture needles. This systematic review and meta-analysis will evaluate the efficacy of electroacupuncture in treating obesity.

**Methods:**

MEDLINE/PubMed, EMBASE, the Cochrane Central Register of Controlled Trials, 3 Korean databases (Oriental Medicine Advanced Searching Integrated System, ScienceON, and KoreaMed), 1 Japanese database (Citation Information by the National Institute of Informatics), and 1 Chinese database (Chinese National Knowledge Infrastructure) will be searched from their inception to December 2021. The primary outcome will be body mass index, and the secondary outcomes will be body weight, waist and hip circumference, waist-to-hip ratio, body fat percentage, body fat mass, and adverse effects.

**Results and conclusion:**

This systematic review and meta-analysis will provide evidence for efficacy of electroacupuncture as a treatment method for obesity.

**Trial registration number:**

DOI 10.17605/OSF.IO/YU5XR (https://osf.io/yu5xr).

## 1 Introduction

Obesity is a public health problem of the modern society that negatively affects the quality of life and leads to health conditions. The prevalence of obesity and overweight continues to increase regardless of sex or region, and has approximately doubled since 1980.^[1]^ Obesity is associated with an increased risk of cardiovascular diseases,^[2]^ diabetes,^[3]^ cancer,^[4]^ and depression.^[5]^ It is known to reduce life expectancy.^[6,7]^

Various treatment strategies have been studied and applied for obesity. Lifestyle interventions in the form of diet control, physical exercise, and behavioral modifications have also been developed.^[8]^ If they are ineffective, drugs or surgical intervention should be considered. However, drugs lead to gastric, psychiatric as well as cardiovascular problems^[9]^ and surgical interventions cause anemia and lead to reoperations.^[10]^ Accordingly, patients are advised drug treatment and surgery based on strict standards. Although the number of patients with obesity is increasing, treatment options are limited. Therefore, there is a growing interest in safe and effective treatments having limited adverse effects.

Electroacupuncture is a type of acupuncture, which involves the transmission of electrical stimulation to the body through acupuncture needles. It is thought that electroacupuncture strengthens stimulation at acupoints as compared with acupuncture.^[11]^ Anti-obesity effects of electroacupuncture have been studied^[12,13]^ and is widely used to treat obesity in clinical situations. However, only 1 systematic review and meta-analysis, which included articles until 2019, has been published.^[14]^

In this protocol, we present a systematic review and meta-analysis of the efficacy of electroacupuncture as an obesity treatment.

## 2 Methods

### 2.1 Study registration

This protocol is written based on the preferred reporting items for systematic reviews and meta-analysis (PRISMA) guidelines.^[15]^ This protocol is registered in the Open Science Framework (https://osf.io/yu5xr).

### 2.2 Eligibility criteria for study selection

#### 2.2.1 Types of studies

Only randomized controlled trials will be analyzed in this systematic review to obtain high-quality evidence. Animal studies or quasi-randomized controlled trials will be excluded. There will be no limitation on type of language in the selection process.

#### 2.2.2 Types of participants

Patients diagnosed with simple obesity will be included in this study. Participants will not be restricted based on age, sex, or race.

#### 2.2.3 Types of interventions

Studies with the experimental group treated with electroacupuncture for simple obesity will be included. Electroacupuncture with usual care including physical exercise, diet control, and dietary education will also be included as an intervention in this review. Electroacupuncture combined with other treatments including moxibustion, cupping, and catgut embedding therapies will be excluded. Control groups will include untreated patients and those in usual care.

#### 2.2.4 Types of outcome measures

The primary outcome will be body mass index. The secondary outcomes will be body weight, waist circumference, hip circumference, waist-to-hip ratio, body fat percentage, body fat mass, and adverse effects.

### 2.3 Search strategy

#### 2.3.1 Electronic data

We will search the following 8 electronic databases from their inception to December 2021 for this review: MEDLINE/PubMed, EMBASE, the Cochrane Central Register of Controlled Trials, 3 Korean databases (Oriental Medicine Advanced Searching Integrated System, ScienceON, and KoreaMed), 1 Japanese database (Citation Information by the National Institute of Informatics), and 1 Chinese database (Chinese National Knowledge Infrastructure). The search strategy for MEDLINE/PubMed is presented in Table 1.

**Table 1:** Search strategy for PubMed.

(“obes∗”[All Fields] OR “weight gain∗”[All Fields] OR “weight loss”[All Fields] OR “body mass ind∗”[All Fields] OR “adipos∗”[All Fields] OR “overweight”[MeSH Terms] OR “overweight”[All Fields] OR “overweighted”[All Fields] OR “overweightness”[All Fields] OR “overweights”[All Fields] OR “over weight”[All Fields] OR “overload syndrome∗”[All Fields] OR “overeat∗”[All Fields] OR “over eat∗”[All Fields] OR “overfeed∗”[All Fields] OR “over feed∗”[All Fields] OR “weight cycling”[All Fields] OR “weight reduc∗”[All Fields] OR “weight los∗”[All Fields] OR “weight maint∗”[All Fields] OR “weight decreas∗”[All Fields] OR “weight chang∗”[All Fields]) AND (“electroacupuncture”[MeSH Terms] OR “electroacupuncture”[Text Word] OR “electro-acupuncture”[Text Word] OR “electric acupuncture”[Text Word]) AND (“randomized controlled trial”[All Fields] OR “controlled clinical trial”[All Fields] OR “random∗”[All Fields] OR “controlled”[All Fields] OR “placebo”[All Fields] OR “trial”[All Fields])

#### 2.3.2 Search for other resources

For a wider review of related articles, the reference lists of the included articles will be scanned. We will also manually search for offline articles.

### 2.4 Data collection and analysis

#### 2.4.1 Study selection

**Figure 1:**
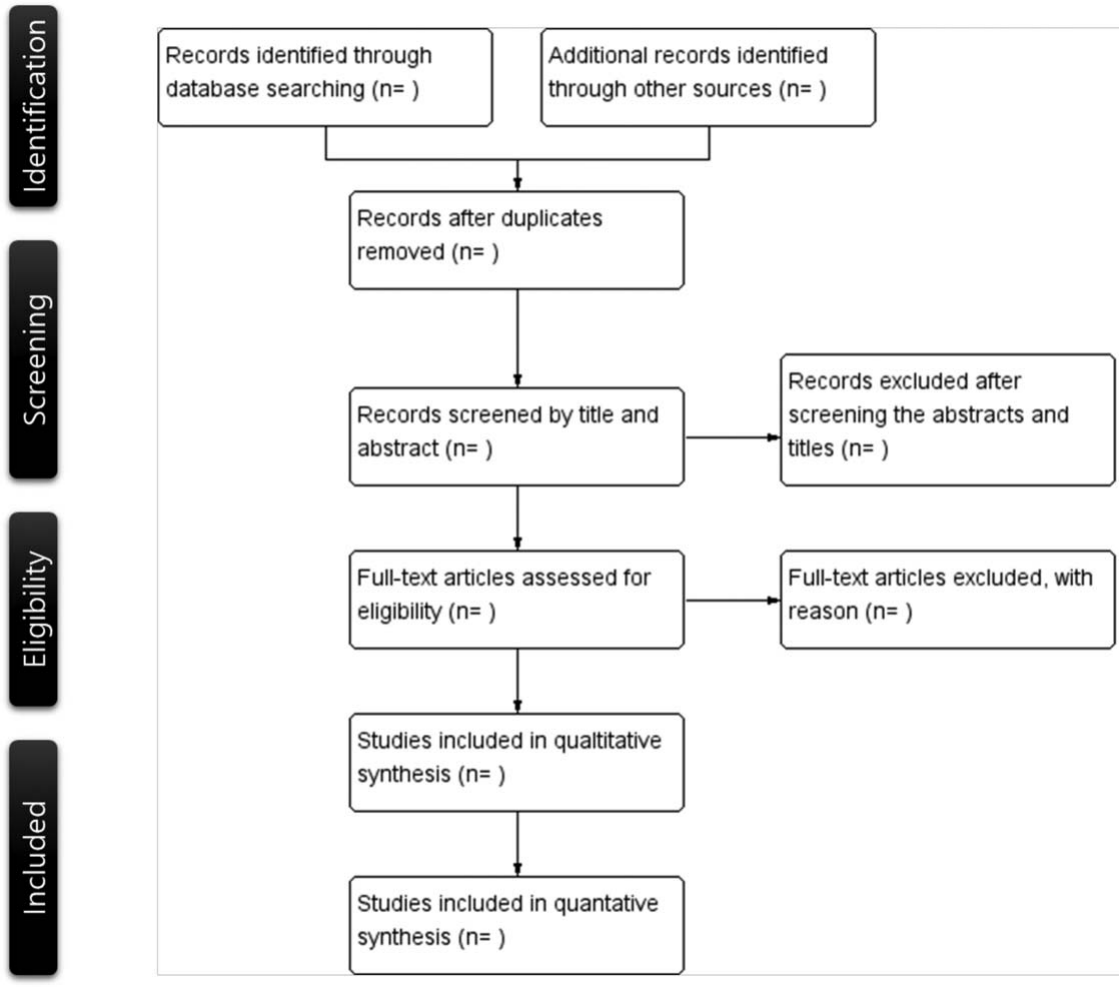
Flow diagram of the systematic review.

According to the search guidelines, 2 independent researchers will search electronic databases and other resources individually. The titles, abstracts, and main texts of the articles will be scanned. Differences between the 2 researchers will be resolved through a discussion with a third researcher. The final decision will be made by the third researcher. A flow diagram of the search process is shown in Figure 1.

#### 2.4.2 Data extraction and management

Two researchers will use an Excel spreadsheet for data extraction. The following items will be listed in the Excel spreadsheet: author names, publication year, study design, details of the experimental and control groups, number of participants in each group, duration and frequency of treatment, results of outcome measures, and adverse effects. If the Excel spreadsheets do not match between the 2 researchers, a third researcher will make the final decision.

#### 2.4.3 Assessment of the risk of bias and quality of the included studies

The risk-of-bias tool developed by the Cochrane collaboration group will be used to evaluate the risk of bias.^[16]^ The domains of risk-of-bias include random sequence generation, allocation concealment, blinding of participants and personnel, blinding of outcome assessment, incomplete outcome data, selective outcome reporting, and other sources of bias. Each domain will be evaluated in 3 categories: high risk, low risk, and unclear risk. If the assessments for the risk of bias between 2 researchers are different, a third researcher will make the final decision.

#### 2.4.4 Assessment of the effect of treatment

Continuous outcome data will be presented as mean differences with 95% confidence intervals.

#### 2.4.5 Management of missing data

If necessary data are missing in the article, the corresponding author will be contacted for more information. If the data cannot be received, the data will not be included in the analyses.

#### 2.4.6 Data synthesis

The extracted data will be synthesized using Review Manager software version 5.4, distributed by the Cochrane collaboration. Statistical heterogeneity among studies will be assessed using Higgins I^2^ statistic. A random-effects model will be used in consideration of heterogeneity because this review will include studies conducted in various countries without restrictions on age, gender, or race.

#### 2.4.7 Subgroup analysis

Subgroup analysis will be conducted according to the type of experimental group and control groups.

#### 2.4.8 Ethics and dissemination

As this is a protocol for the study of systematic review and meta-analysis, ethical approval is not required. The results and conclusions of this systematic review and meta-analysis will be published and submitted to an academic journal to disseminate the findings.

## 3 Discussion

Obesity is a chronic health problem associated with various diseases, and its prevalence is on the rise in recent times. Studies on the effects and mechanisms of electroacupuncture for obesity have been steadily researched, and it is widely used in clinical situations. This systematic review and meta-analysis will provide evidence for the efficacy of electroacupuncture as a treatment for obesity. The results of this study would be helpful for patients with obesity as well as clinicians.

## Author contributions

**Conceptualization:** Junhyuk Kang, Koh-Woon Kim, Yeonho Seo, Mi-Yeon Song, Won-Seok Chung.

**Data curation:** Junhyuk Kang, Koh-Woon Kim, Yeonho Seo.

**Formal analysis:** Junhyuk Kang, Koh-Woon Kim, Mi-Yeon Song.

**Funding acquisition:** Mi-Yeon Song, Won-Seok Chung.

**Project administration:** Junhyuk Kang, Koh-Woon Kim, Yeonho Seo, Mi-Yeon Song, Won-Seok Chung.

**Writing – original draft:** Junhyuk Kang, Koh-Woon Kim, Yeonho Seo.

**Writing – review & editing:** Junhyuk Kang, Mi-Yeon Song, Won-Seok Chung.

## References

[1] Chooi YC Ding C Magkos F. The epidemiology of obesity. Metabolism 2019;92:06–10. 10.1016/j.metabol.2018.09.005 30253139

[2] Koliaki C Liatis S Kokkinos A. Obesity and cardiovascular disease: revisiting an old relationship. Metabolism 2019;92:98–107. 30399375 10.1016/j.metabol.2018.10.011

[3] Nguyen NT Nguyen XM Lane J Wang P. Relationship between obesity and diabetes in a US adult population: findings from the National Health and Nutrition Examination Survey, 1999–2006. Obes Surg 2011;21:351–5. 21128002 10.1007/s11695-010-0335-4 PMC3040808

[4] Steele CB Thomas CC Henley SJ . Vital signs: trends in incidence of cancers associated with overweight and obesity—United States, 2005–2014. MMWR Morb Mortal Wkly Rep 2017;66:1052–8. 28981482 10.15585/mmwr.mm6639e1 PMC5720881

[5] Luppino FS de Wit LM Bouvy PF . Overweight, obesity, and depression: a systematic review and meta-analysis of longitudinal studies. Arch Gen Psychiatry 2010;67:220–9. 20194822 10.1001/archgenpsychiatry.2010.2

[6] Chang SH Pollack LM Colditz GA. Life years lost associated with obesity-related diseases for U.S. non-smoking adults. PLoS One 2013;8:e66550. 23823705 10.1371/journal.pone.0066550 PMC3688902

[7] Fontaine KR Redden DT Wang C Westfall AO Allison DB. Years of life lost due to obesity. JAMA 2003;289:187–93. 12517229 10.1001/jama.289.2.187

[8] Webb VL Wadden TA. Intensive lifestyle intervention for obesity: principles, practices, and results. Gastroenterology 2017;152:1752–64. 28192109 10.1053/j.gastro.2017.01.045

[9] Muller TD Bluher M Tschop MH DiMarchi RD. Anti-obesity drug discovery: advances and challenges. Nat Rev Drug Discov 2021;01–23. 10.1038/s41573-021-00337-8 PMC8609996 34815532

[10] Gloy VL Briel M Bhatt DL . Bariatric surgery versus non-surgical treatment for obesity: a systematic review and meta-analysis of randomised controlled trials. BMJ 2013;347:f5934. 24149519 10.1136/bmj.f5934 PMC3806364

[11] Fu L Zhong J Fu Q Yang Y Zhang M Zhang Q. Clinical effects and safety of electroacupuncture for the treatment of allergic rhinitis: a protocol for systematic review. Medicine (Baltimore) 2020;99:e18931. 32028401 10.1097/MD.0000000000018931 PMC7015649

[12] Lu M He Y Gong M . Role of neuro-immune cross-talk in the anti-obesity effect of electro-acupuncture. Front Neurosci 2020;14:151. 32180699 10.3389/fnins.2020.00151 PMC7059539

[13] Wang F Tian DR Han JS. Electroacupuncture in the treatment of obesity. Neurochem Res 2008;33:2023–7. 18719995 10.1007/s11064-008-9822-6

[14] Gao Y Wang Y Zhou J Hu Z Shi Y. Effectiveness of electroacupuncture for simple obesity: a systematic review and meta-analysis of randomized controlled trials. Evid Based Complement Alternat Med 2020;2020:2367610. 32714399 10.1155/2020/2367610 PMC7341404

[15] Shamseer L Moher D Clarke M . Preferred reporting items for systematic review and meta-analysis protocols (PRISMA-P) 2015: elaboration and explanation. BMJ 2015;350:g7647. 25555855 10.1136/bmj.g7647

[16] Higgins JP Altman DG Gotzsche PC . The Cochrane Collaboration's tool for assessing risk of bias in randomised trials. BMJ 2011;343:d5928. 22008217 10.1136/bmj.d5928 PMC3196245

